# Coordinated patterns of gene expressions for adult muscle build-up in transgenic mice expressing myostatin propeptide

**DOI:** 10.1186/1471-2164-10-305

**Published:** 2009-07-08

**Authors:** Baoping Zhao, Eileena J Li, Robert J Wall, Jinzeng Yang

**Affiliations:** 1 Dept of Human Nutrition, Food and Animal Sciences, University of Hawaii at Manoa, 1955 East West Road, Honolulu, HI 96822, USA; 2 Animal and Natural Resources Institute, USDA-ARS, Beltsville, MD 20705, USA

## Abstract

**Background:**

Skeletal muscle growth and maintenance are essential for human health. One of the muscle regulatory genes, namely myostatin, a member of transforming growth factor-β, plays a dominant role in the genetic control of muscle mass. Myostatin is synthesized as a precursor protein, which generates the N-terminal propeptide and the C-terminal mature myostatin peptide by a post-translational cleavage event. Previously, transgenic over-expression of myostatin propeptide in skeletal muscle results in significant muscle growth in early stages of development. The objectives of present study were to further characterize muscle growth in later stages of life and to identify genes and their expression patterns that are responsible for adult muscle build-up by myostatin propeptide.

**Results:**

Immunohistochemical staining with an antibody to the N-terminus indicates a high level of myostatin propeptide present in the muscles of transgenic mice while there were no apparent differences in myostatin protein distribution in the muscle fibers between the transgenic and wild-type mice. Main individual muscles increased by 76–152% in the transgenic mice over their wild-type littermate mice at 12 months of age. A large number of nuclei were localized in the central and basal lamina of the myofibers in the transgenic mice as the number of nuclei per fiber and 100 μm^2 ^area was significantly higher in transgenic mice than wild-type mice. By systemic comparisons of global mRNA expression patterns between transgenic mice and wild-type littermates using microarray and qRT-PCR techniques, we have identified distinct gene expression patterns to support adult muscle build-up by myostatin propeptide, which are comprised of enhanced expressions of myogenic regulatory factors and extracelullar matrix components, and differentially down-regulated expressions of genes related to protein degradation and mitochondrial ATP synthesis.

**Conclusion:**

The results present a coordinated pattern of gene expressions for reduced energy utilization during muscle build-up in adult stage. Enhanced muscle buildup by myostatin propeptide is sustained by reduced ATP synthesis as a result of a decreased activity of protein degradation. Myostatin propeptide may have a therapeutic application to the treatment of clinical muscle wasting problems by depressing myostatin activity.

## Background

Skeletal muscle growth and maintenance are essential for human health. A basic understanding of muscle growth has many clinical applications as it can be used to treat serious muscle-related diseases such as muscular dystrophy and muscle wasting. Muscle fibers elongate and increase in size by fusion of myoblast cells. Myoblasts are rapidly dividing cells in culture, but cease the proliferation and DNA synthesis once they fuse into myotube [1]. In mammals, myofiber numbers are determined before birth, postnatal muscle growth primarily results from elongation or increase in muscle fiber size. In adults, skeletal muscle regenerative properties decline with age. Myostatin, one of the muscle regulatory genes, is a member of the transforming growth factor-β superfamily. It regulates muscle formation during embryogenesis and postnatal muscle development as an endogenous inhibitor of muscle mass. Myostatin mRNA sequences were conserved across most mammalian species. In the absence of myostatin function, massive muscle growth has been observed in mice, cattle and humans [2]. In particular, mice with null mutations in myostatin gene have twice the muscle mass as wild-type mice, resulting from muscle fiber hyperplasia and hypertrophy [3]. Whereas, mice with over-expression of myostatin in skeletal muscle is associated with lower muscle mass and decreased fiber size and increased fat mass [4]. Like other TGF-β family members, myostatin is synthesized as a precursor protein, which undergoes two post-translational cleavage events. The first cleavage event removes the 24-amino acid signal peptide, and the second cleavage, at an RSRR site located at amino acid sequence 240–243, generates an N-terminal and a C-terminal peptide. The N-terminal peptide is referred to as myostatin propeptide while the C-terminal peptide is the actual mature form of myostatin with ligand binding activity. The protenase furin is showed to cleave the RSRR site in CHO cells [5,6]. The precursor protein is detected as a predominant form of myostatin in muscle extracellular matrix and can also be cleaved by furin proteases [7]. Transgenic over-expression of myostatin propeptide in skeletal muscle increases animal growth and muscle mass [5,8]. Enhanced muscle mass phenotype in the propeptide transgenic mice is primarily achieved by myofiber hypertrophy rather than myofiber hyperplasia. The size of fast-twitch, glycolytic muscle fiber at 9 weeks of age was increased by 60% compared with wild-type littermates [8]. Our recent study with the propeptide transgenic mouse model revealed that sufficient muscle growth during adolescence can prevent high-fat diet induced obesity and type II diabetes during adulthood [9].

Myostatin is secreted to the intramuscular spaces in the form of the so-called latent complex. Upon dissociation of the latent complex, myostatin binds to activin receptor type IIB and activates Smad2/3 signaling pathway to inhibit myoblast cell proliferation and differentiation. During cell cycles, cyclin-dependent kinases (Cdks) regulate G_1 _phase transitions to S phase. Myostatin is able to increase Cdk inhibitor p21 activity, therefore decreasing the Cdk levels, concurrently resulting in myoblast cell cycle arrest in the G_1 _phase [10,11]. Myostatin also inhibits MyoD expression and activity via Smad3, which blocks myoblasts from differentiating into myotubes [12,13]. Therefore, myostatin inhibits both myoblast cell proliferation and differentiation. Myostatin is also expressed in satellite cells and adult myoblasts. It negatively regulates the G1 to S progression of satellite cells to maintain their quiescent status [14]. Muscles from myostatin-null mice have an increased number of satellite cells as well as a higher proportion of activated satellite cells than muscles of wild-type mice [14,15]. A study with myostatin short interfering hairpin RNA transfer to rats also showed a significant increase in tibialis anterior weight and fiber size and over 2-fold change of satellite cell number [16]. These results clearly suggest that myostatin maintains satellite cells in a quiescent state in adult muscle.

To further characterize the mechanism by which transgenic expression of myostatin propeptide enhanced muscle mass, we have located the expressions of myostatin and its propeptide proteins and studied muscle fiber formation in adult skeletal muscle. Interestingly, transgenic mice maintained muscle build-up at one year of age. Based on the role myostatin in muscle satellite cells and broad physiological regulation of muscle build-up, we hypothesized that various genes or pathways involved in muscle growth, protein synthesis and degradation, energy supply and utilizations to muscle tissue are responsible for the continuous muscle build-up as a result of myostatin depression by its propeptide. We compared gene expression patterns of skeletal muscle between myostatin propeptide transgenic mice and their littermate wild-type mice. Here, we reported the observations of a clustering of nuclei in the centre of the adult muscle fibers of the transgenic mice. Gene expression patterns were consistent with enhanced muscle build-up, consisting of enhanced myogenic regulatory factors and extracelullar matrix components with down-regulated activities of protein degradation and mitochondrial ATP synthesis. Enhanced muscle build-up in adult stage is sustained by reduced ATP synthesis as a result of a decreased activity of protein degradation. The results present a distinct coordinated pattern of gene expressions for reduced energy utilization during adult muscle build-up by myostatin propeptide.

## Methods

### Animals and Tissue Sampling

**Figure 1:**
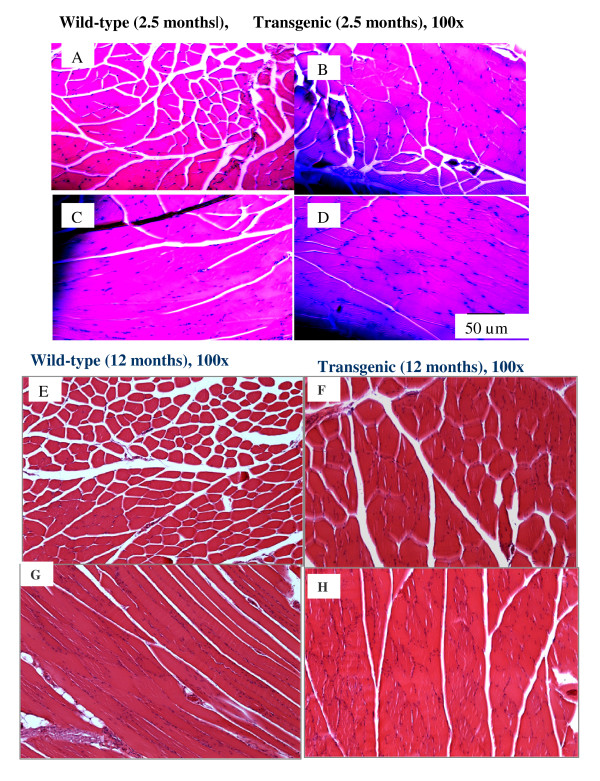
**Comparisons of muscle fiber staining of biceps femoris between wild-type and transgenic mice**. Mice were sacrificed at 2.5 months and 12 months of age. Biceps femoral muscle samples were used for this study, and samples were frozen-sectioned. Hematoxylin and Eosin Staining of both wild-type (A, C, E, G, I, K) and transgenic muscle samples (B, D, F, H, J, L) shows the myofiber size and nuclei. Panels of C, D, G, H was longitudinally sectioned to show myofiber nuclei. Wild-type mice (C and G) in young and adult ages maintained relative less myofiber nuclei while transgnic mice (D and D) showed accumulative nuclei. Arrows point the formation of myoblast nuclei (J, L) of transgenic mice in adult age from satellite cells and myofiber fusions in comparisons with wild-type mice (I, K).

Myostatin propeptide-transgenic mice were generated by standard microinjection techniques, which has been previously described [8]. Male mice (hemizygous genotype for the transgene) from the high-expressing line were mated with B6SJL F1 wild-type females to produce offspring mice, which were used in this study. Mice were housed in cages; room temperature was maintained at 22°C and 12-h light/dark cycle. Mice were weaned at 4 weeks of age, and given free access to a chow diet (10% kcal fat, ME3.85 kcal/g). All animal experiments were approved by the Institutional Animal Care and Use Committee of the University of Hawaii. Male mice at 2.5 months and 12 months of age were sacrificed for muscle tissue dissections and sampling after an overnight fasting. Gastrocnemius and biceps femoral muscle samples and the white portion of the muscles were immediately dissected from carcass, cleaned from fat, blood and quickly frozen in liquid nitrogen, and later stored in a -80°C freezer. Tissue samples from 12 month old mice were used for all the studies expect the muscle histology studies in Figure 1, which used tissue from both 2.5 and 12 month old mice.

### Immunohistochemistry

Gastrocnemius and biceps femoral muscle samples of three transgenic and three wild-type littermate mice were used for this study. Muscle samples was sectioned and transferred to impregnated slides in ice-cold acetone prior staining. After quenching in 0.3% H_2_O_2_, the sections were blocked in 10% rabbit serum, 1% BSA for 30 min and incubated with or without the myostatin C-terminal antibody (1:250, Santa Cruz Biotech) overnight. Then the sections were incubated with an HRP-coupled secondary antibody (1:250) followed by a cell nuclei counterstaining with diaminobenzidine (DAB)/peroxidase reaction (0.05 mg/ml DAB, 0.006% H_2_O_2_) until the color was developed. For immuno-fluorescence double labeling of myostatin propeptide, muscle samples were extracted, fixed, embedded, cut, deparaffinized and re-hydrated. Afterwards, they underwent three PBS washings, a 30 min incubation with 3% NGS in PBS in the moist chamber, and 1:50 dilution of primary antibody (GDF-8, N19, Santa Cruz Biotech) in 3% NRD for 2 hours. They were washed in TPBS and covered with rabbit anti-goat IgG (Santa Cruz Biotech) with 1:300 dilution for 30 min at room temperature. After washing in PBS, coverslips with P2 counterstain mounting medium was applied. The sections were then mounted on glass slides and coverslipped using Gelovatol. The sections were examined using Zeiss LSM5 laser-scanning confocal microscope. All comparisons of staining intensity between transgenic and wild-type mice were done on sections stained simultaneously and the imaging for each antibody was performed using identical laser power and software settings to ensure validity of intensity comparisons.

### Hematoxylin and Eosin Staining

Wild-type and transgenic muscle samples were prepared as mentioned in the previous procedures. After re-hydration, they were placed in distilled water for 5 min followed by 16 h dips in hematoxylin, and further washed for 30 sec. 1% Eosin was added for 1 min, then 1% glacial acetic acid for another 1 min. The samples were washed in tap water until the red stain turned light red. Dehydration using absolute alcohol was followed by a 5 min xylene rinse before mounting with pertex.

### Urine sampling and Assays for Urinary Creatinine and 3-Methylhistidine

Urine samples were collected several times in the mornings from 8 transgenic and 8 wild-type littermate mice at 12 months of age. Creatinine concentration was determined by the standard picric acid method [17], and 3-methylhistidine concentration was determined on deproteinized samples using automatic amino acid analysis (Backman Model 6300) following the method [18].

### Preparation of Total RNA

Total RNA was isolated from gastrocnemius muscle tissues using TRIzol reagent (Invitrogen, Carlsbad, CA) and chloroform. Approximately 100-mg muscle tissue was used for the total RNA extraction and prepared on dry ice. Tissue was then homogenized in TRIzol reagent using a Polytron homogenizer at maximum speed for 60 seconds. Concentration of total RNA was determined by measuring absorbance at 260 and 280 nm using a Smart Spec 3000 (BioRad, Hercules, CA). The RNA purity and quality was determined by ratio of A260 to A280 and confirmed by Agilent 2100 Bioanalyzer Analysis with the RNA 600 Nano Assay kit (Agilent Technologies).

### Preparation of cRNA and Gene Chip Hybridization

Total RNA from each group of transgenic and wild-type mice (n = 5) was pooled in equal molar amount before use for the experiment with GeneChip Mouse Genome 430 2.0 Arrays (Affymetrix, Santa Clara, CA), which contain probes for detecting 45,000 transcripts with over 34,000 well-characterized genes. After RNA isolation, all the subsequent technical procedures including quality control and concentration measurement of RNA, cDNA synthesis and biotin-labeling of cRNA, hybridization and scanning of the arrays were performed at the Stanford Functional Genomics Facility (Stanford, CA). Each chip was used for pooled total RNA from five mice per treatment group. Briefly, prior to the reverse transcriptase reaction, RNA was treated with deoxyribonuclease I (Invitrogen) to remove any residual genomic DNA. cDNA was then synthesized with 5 μg total RNA by SuperScript II reverse transcriptase (Invitrogen) and purified by phenol/chloroform extraction. Then cDNA was labeled using the ENZO BioArray RNA transcript labeling kit (Enzo Life Sciences, Inc., Farmingdale, NY, USA) to generate biotinylated cRNA. Biotin-labeled cRNA was purified and fragmented according to Affymetrix's protocol. The fragmented cRNA was mixed with control oligonucleotide B2 (Affymetrix) and a hybridization control cRNA mixture (BioB, BioC, BioD, and Cre, Affymetrix). Chips were hybridized at 45°C for 16 h. The arrays were subsequently washed and stained in a Fluidics Station (Affymetrix) and scanned by GeneScanner 3000 according the manufacturer's instructions (GeneChip Expression Analysis Technical Manual, Affymetrix).

### Quantitative real-time PCR

Quantifications of mRNA levels for selected genes were performed by Quantitative real-time-polymerase chain reaction (qRT-PCR) with SYBR Green reagent in an ABI 7300 Sequence Detection System (Applied Biosystems, Forrest City, CA). Primers were designed by Primer Express 3.0 software (Applied Biosystems, Foster, CA) and listed in Table 1. The qRT-PCR was performed on an extended set of sample of 4 transgenic mice and 4 wild-type mice in addition to the RNA of the pooled samples used in microarray analysis. Optimal annealing temperatures for the primers used were determined to be 60°C and 45 cycles. The abundance of each mRNA transcript was measured and expressed in comparison to glyceraldehyde 3-phosphate dehydrogenase (GAPDH). The GAPDH expression was not different between transgenic and wild-type mice. Relative expression of mRNA was determined by calculations of threshold cycle (Ct) according to protocol set by Applied Biosystems, and expressed as fold change compared to wild-type mice. Fold change was calculated by subtracting the Ct number of the gene of interest from the Ct of the endogenous GAPDH. The result of this calculation was termed ΔCt, which was further used for calculation of ΔΔCt of each transgenic mouse based on the mean value of the wild-type mice. The fold change was calculated as Log_10_2^(-ΔΔCt) ^[19].

**Table 1:** Primer sequences used for qRT-PCR

Target Gene	Accession No.	Amplicon (bp)	Forward Primer (5-3)	Reverse Primer (5-3)
Glyceraldehyde-3-phosphate dehydrogenase	NM_199472	191	aacgaccccttcattgac	tccacgacatactcagcac
Myostatin propeptide	U84005	99	gctctttggaagatgacgat	catttgggcttgccatcc
Myogenin	NM_031189	100	actcccttacgtccatcgtg	acccagcctgacagacaatc
Follistatin-like 1	NM_008047	96	cagccaggaatagcatggat	ctcttcctgggcagagtgac
Cyclin-dependent kinase inhibitor 1A	NP001104569.1	117	ttgggaaggaaaagggctat	gaggaaccgtccaagaatga
Procollagen, type I, alpha 1	U08020	110	gacctcagggtattgctgga	accttgtttgccaggttcac
Procollagen, type I, alpha 2	AW545978	138	atgcacatcaatgtggagga	aggctgacacgaactgaggt
Procollagen, type III, alpha 1	BG968894	96	ctatgacattgggggtcctg	ttttgttttgctggggtttc
Procollagen, type V, alpha 2	NM_007737	111	gcagctccagatgacacaaa	tgggtgtttcttggaaccat
Procollagen, type V, alpha 3	NM_016919	99	gctcttctgtgggtttcctg	taaagcagatggagccgagt
Procollagen, type VI, alpha 1	NM_009933	109	tgacccaactggtcaactca	gggcgggatctagataggag
Procollagen, type VI, alpha 2	BI455189	106	aacccaaagccccttaccta	agactctggggtcctccaat
Procollagen, type VI, alpha 3	AF064749	101	acggagaacagtgccagact	agaaccaaggactggtcgtg
Fibronectin 1	BC004724	114	agtgcttcatgccgctagat	acatcactggggtgtggatt
Biglycan	BC019502	93	ggtgggcatcaatgacttct	cagtagggcacagggttgtt
Calpain 3	AI323605	88	ccaccctaaaagtggcagaa	ctgggttgtccatagcacct
Calpain 7	BG068214	93	agtccccatgatgaaagcac	gcaggttggtgaatgtagca
Caspase 7	BB752393	110	cctggcactattggggtaaa	gccatcaaaaagggacacat
Ubiquitin specific protease 25	NM_013918	126	cttcccagggtcaccataga	ggtcggcatagtcgttttgt
Atrogin 1/F-box protein 32	NM_026346	115	gttttcagcaggccaagaag	ttgccagagaacacgctatg
Ubiquitin-conjugating enzyme E2D 3	BG070073	147	gtgacttgcattgggttcct	tgatcatgctgtgttcgtga
WW domain E3 ubiquitin protein ligase	BB397174	101	ttggtaggccacactgtcaa	taggagaaagctgggggtct
Proteasome 26S subunit, ATPase, 6	NM_025959	106	acactggatcctgctttgct	gtcctgcgtggattttcaat
Proteasome activator subunit 4	BM195254	99	agtgtggttgagcgtgtcag	agttttgaccgccttgtgtc
NADH dehydrogenase 1 α subcomplex, 1	BC018422	97	gaagtgccctgctttatgga	cgtggaatcctggagatcat
NADH dehydrogenase 1 α subcomplex, 4	BC011114	99	tattggagcagggggtactg	catggctctgggttgttctt
NADH dehydrogenase 1 α subcomplex, 5	NM_026614	108	tctggcaaggaaaatgttga	ccatccaccatctgacactg
NADH dehydrogenase 1 α subcomplex, 7,	C88880	112	gctgccttcatcctgacatt	gcaggccttgaactcagaac
NADH dehydrogenase Fe-S protein 1	BC006660	98	gtgttgctgcagagtggaaa	aatcgcttctaccccaggtt
Ubiquinol-cytochrome c reductase subunit	NM_025650	136	gccttacatcaacggcaagt	ctccagtgtccagcttcctc
Cytochrome c oxidase, subunit Vic	AV111078	115	tcgaagagatgacgaaggcta	atagttcaggagcgcaggtc
ATP synthase mitochondrial F1 complex assembly factor 1	BB771055	99	cccttcagagttgccttcag	gggccataagcgacagttta
ATP synthase, H+ transporting, mitochondrial F1 complex, O subunit	AV066932	96	gaagtgccatgcacagtgac	ttggtttggactcaggaagc
Adrenodoxin	D43690	105	ggaacgttggcttgctctac	aaaagccaggtcaagcatgt
Electron transferring flavoprotein, alpha polypeptide	BC003432	106	tgacaaaaagtgaccgacca	tctgccaggtcatacagcag

### Statistical Analysis

To calculate the number of nuclei, we randomly selected 10 microscopic fields under 40× magnification for both wild-type and transgenic mice. The number of nuclei in the center and the basal lamina for each muscle fiber of bicep muscles were counted. The average number of nuclei per 100 um^2 ^of wild-type and transgenic mice were calculated by an imagine software program. Mean comparisons for muscle fiber nuclei were analyzed by using the JMP program (SAS Inst., Cary, NC). Significant difference between transgenic and wild-type mice was analyzed by two-tailed Student's t-test. Least square means and their standard errors of mean are reported. In the microarray data analysis, significance was determined at p < 0.005, the expression value for each transcript was determined by calculating the average of differences in intensity (perfect match intensity minus mismatch intensity) between its probe pairs. The expression analysis file created from each sample was imported into Affymetrix GeneChip Operating Software (GCOS) for further data characterization. All expression values for genes in the GCOS absolute analyses were determined by global scaling option. The arrays were normalized by quantile normalization and robust multi-array average (RMA) procedure as low level analysis. Perfect- match values were background adjusted, normalized using invariant set normalization and log transformed. The intensities were transformed to log_2 _format and the means of log_2 _were calculated. The mean log_2 _fold change of transgenic group verse the wild-type littermate group was calculated by subtracting the mean log_2 _intensity of wild-type from the mean log_2 _intensity of transgenic group. Statistical significance of the difference in expression levels was determined by two-tailed student's t-test. A transcript was considered differentially expressed if the mean absolute fold change was larger than 1.0 and the p-value was less or equal to 0.005, with mean intensity in the group showing highest expression of larger than 75.

## Results

### Detection of Myostatin and Its Propeptide in Skeletal Muscle

**Figure 2:**
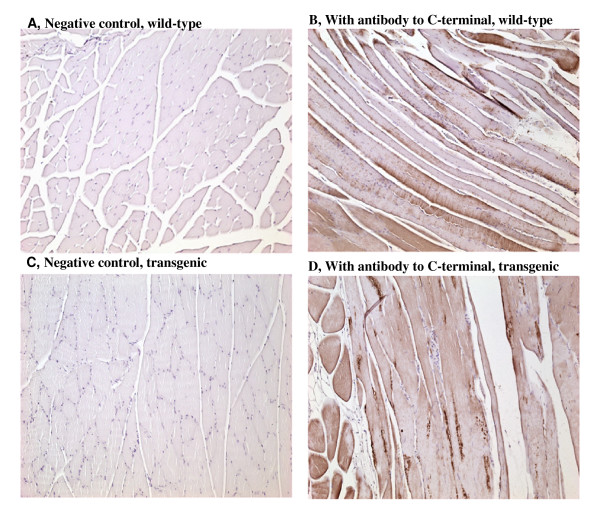
**Localization of myostatin in biceps femoris by immunohistochemistry with antibody to the C-terminal**. Mice were sacrificed at 12 months of age. Biceps femoral muscle samples were used for this study. The cross-sectional area of the muscle samples was frozen-sectioned. The sections were blocked in 10% rabbit serum and incubated with or without (negative control, A and C) the myostatin C-terminal antibody, followed by incubation with an HRP-coupled secondary antibody and counterstaining with diaminobenzidine (DAB)/peroxidase reaction (B and D). Panel A and C show the myofibers which were cross-sectioned, and B and D show myofibers which were longitudinally sectioned. Nuclei were stained as purple/blue color, and myostatin protein was stained as brown color.

**Figure 3:**
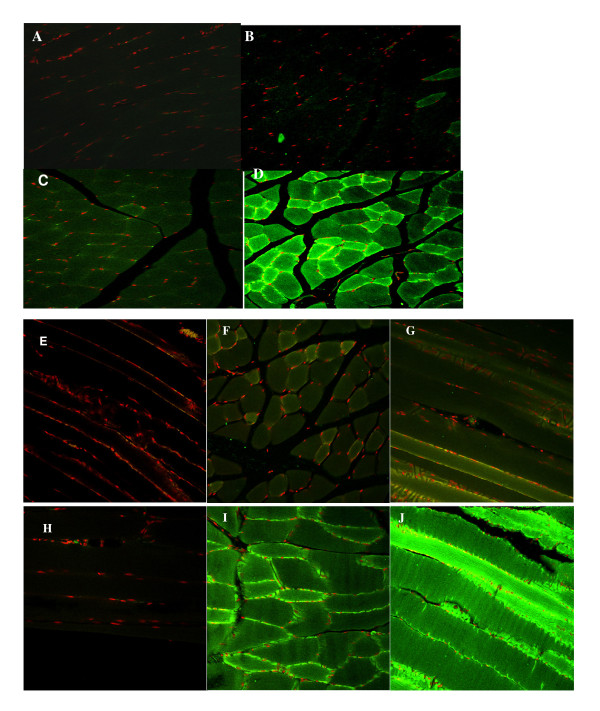
**Detection of myostatin propeptide by immunohistochemistry with antibody to the N-terminal**. Mice were sacrificed at 12 months of age. Gastrocnemius (A to D) and biceps femoral (E to J) muscle samples were used for this study. Frozen muscle samples were cross-sectioned. Following the procedures of immuno-fluorescence double labeling, the sections were incubated with or without (negative control: A, C, E and H) primary antibody to myostatin propeptide. The P2 counter-stain mounting medium was applied, and the images were examined using Zeiss LSM5 laser-scanning confocal microscope. The nuclei were stained as red color, myostatin propeptide reactive with the primary antibody was showed as green color. A (wild-type) and C (transgenic): negative control (without primary antibody), showing myofibers and stained nuclei of Gastrocnemius. B: Gastrocnemius muscle section from wild-type mice was incubated with primary antibody, showing little myostatin propeptide on myofiber surface. D: Gastrocnemius muscle section from transgenic mice was incubated with primary antibody, showing intense myostatin propeptide on myofibers. E (wild-type) and H (transgenic): negative control (without primary antibody), showing myofiber and stained nuclei in cross-section of biceps femoral muscle. F and G: biceps femoral muscle cross-(F) and longitudinal (G) sections from wild-type mice, showing minimal reactive proteins to the primary antibody. I and J: biceps femoral muscle cross-(I) and longitudinal (J) sections from transgenic mice, showing strong and intense density of myostatin propeptide.

The mRNA expressions of myostatin and the transgene propeptide in skeletal muscle has been previously reported [20]. To confirm the presences of the corresponding proteins in skeletal muscle tissue, we employed immunohistochemistry to detect myostatin and the transgene product-propeptide in muscle tissue. By using the antibody to the C-terminus of mouse myostatin, we were able to localize myostatin protein on the myofibers of *Biceps femoris *in both the propeptide transgenic mice and wild-type littermates. There were no apparent differences in myostatin protein distribution in the muscle fibers between the transgenic and wild-type mice (Figure 2). The HRP binding was observed mostly on the surface, as well as the inside of the myofibers in both types of mice although the densities of the stained proteins were not evenly distributed among muscle fibers. In contrast, a clear difference in intensity was observed when an antibody to the N-terminus was used for immunohistochemistry staining of myostatin propeptide in both *Gastrocnemius *and *biceps femoris *of transgenic mice and wild-type mice (Figure 3). Myostatin propeptide, with weak signals or hardly visible in the wild-type mice, stained very intensely in the transgenic mice. Certain fibers from *biceps femoris *of the transgenic mice showed extremely strong staining signals. The same antibodies were previously failed to detect specific proteins by Western blot. These results from Immunohistochemistry indicate a high level of myostatin propeptide present in the muscles of transgenic mice.

### Enhanced Muscle Mass and Fiber Formation in Adult Mice

**Figure 4:**
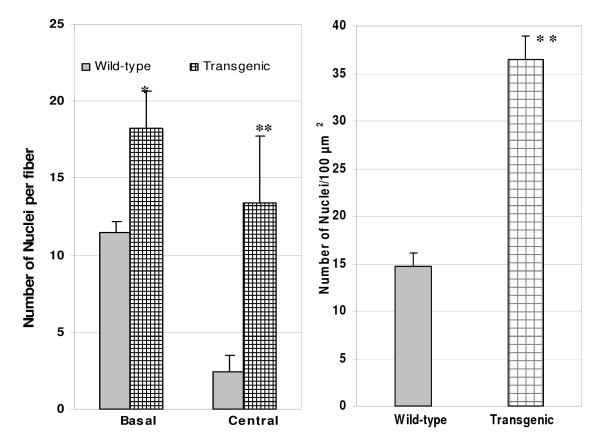
**Comparison of number of myofiber nuclei between wild-type and transgenic mice**. Biceps femoral muscle samples of three transgenic and three wild-type littermate mice were used for this study. Ten random microscopic fields under 40× magnification were selected for both wild-type and transgenic mice. The number of nuclei in the center and the basal lamina for each muscle fiber bicep muscles were counted. The average number of nuclei per 100 um^2 ^were calculated by an image analysis software. Bars represent mean ± SEM (n = 10 per group). Superscript ** and *above the bars indicate significant differences at P < 0.01 and P < 0.05, respectively.

At 12 months of age, mice were sacrificed and muscle tissues were dissected. Muscle weights from transgenic and wild-type littermate mice are summarized in Table 2. Main muscles from transgenic mice weighed significantly more than those from wild-type littermates. The percentage increase in main muscles of transgenic mice over the wild-type mice ranged from 76% to 152%. To characterize the nature of the consistent muscle enhancement, we further characterized the muscle fiber histology (Figure 1). The muscle fiber staining from young animals at 2.5 months of age confirmed our previous observation that transgenic mice showed increased fiber size and muscle fusions [20]. Interestingly, detailed observations of the myofiber histology indicated more nuclei were localized in the central and basal lamina of the myofibers of the transgenic mice (Figure 1) although these mice were at one year of age. Long and stretched nuclei were also noted in some fibers, suggesting active fiber fusion in these muscles. In contrast, muscle histology from wild-type littermates did not show such changes in nuclei distributions. The number of nuclei per fiber in both basal and central lamina of the myofiber were significantly higher in transgenic mice than in wild-type littermates by 58.3% and 458%, respectively (P < 0.01, Figure 4). Similarly, the number of nuclei per 100 μm^2 ^fiber area was also higher by 149% in transgenic mice than in wild-type littermates (P < 0.01, Figure 4) at 12 months of age. These results provide evidences that transgenic expression of myostatin propeptide supported continuous muscle build-up in adult skeletal muscle tissue.

**Table 2:** Individual muscle weight of transgenic and wild-type littermate mice*

**Individual Muscle**	**Wild-type**	**Transgenic**	**% increase of wild-type**
Observations	12	11	

Body weight	31.2 ± 1.27^a^	38.6 ± 0.57^b^	24

Gastrocnemius/plantaris	0.25 ± 0.035^a^	0.53 ± 0.026^b^	112

Biceps femoris	0.27 ± 0.023^a^	0.68 ± 0.038^b^	152

Semitendinosus	0.31 ± 0.022^a^	0.72 ± 0.046^b^	132

Pectoralis	0.32 ± 0.029^a^	0.49 ± 0.015^b^	53

Longissimus dorsi	0.44 ± 0.019^a^	0.80 ± 0.045^b^	82

Triceps brachii	0.29 ± 0.028^a^	0.51 ± 0.033^b^	76

*Transgenic and wild-type littermate mice were sacrificed at 12 months of age. Individual muscles were dissected and weighed at the time of sacrifice. Means ± SEM with different superscript letter were different at P < 0.01.

### Microarray Analysis of Global mRNA Expression in Adult Skeletal Muscle

**Figure 5:**
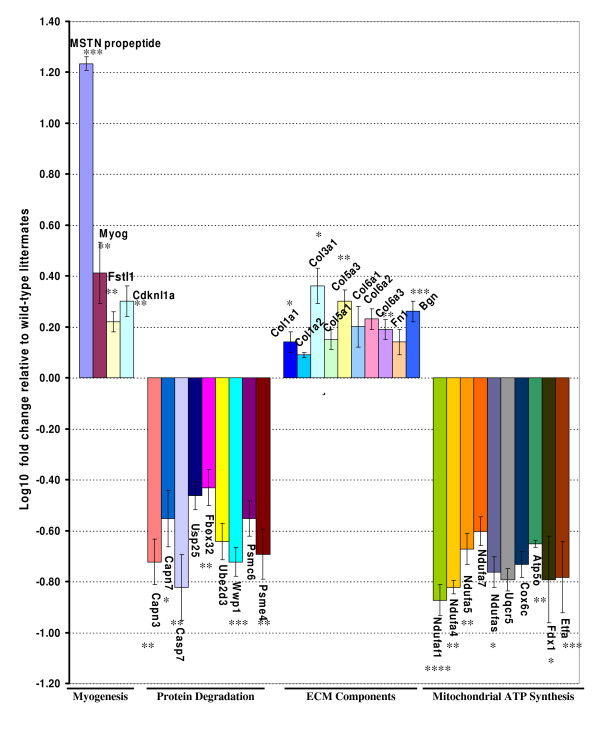
**Expression analysis by qRT-PCR**. The mean and SEM at each bar represents the Log_10 _fold change of transgenic mice relative to their wild-type littermate mice. Statistical differences between transgenic and wild-type mice were determined by two-sided Student's t test (*: P < 0.05; **: P < 0.01; ***: P < 0.001). The gene symbols can be referred to Table 2.

To undercover molecular and global dynamic changes of skeletal muscle enhancements and metabolisms resulting from transgenic expression of myostatin propeptide in the adult stage, we obtained global gene expression profiles of gastrocnemius muscle by microarray analysis. The results from microarray data analysis indicated 52 unique genes differentially expressed in the adult muscle of the transgenic mice at the statistical significance of P < 0.005. A cluster analysis of these genes indicates several functional categories, including genes closely related to myogenesis, extracellular matrix components, protein degradation, mitochondrial ATP synthesis, and carrier proteins. The expression levels of specific mRNA were presented as mean log_2 _fold change of transgenic group compared with the wild-type littermates (Table 3). The expression of the genes related to myogenesis and extracellular matrix formation were differentially increased by the propeptide transgene, with fold change from 1.9 to 3.7 at P < 0.005, while the expression of the genes corresponding to protein degradation, mitochondrial ATP synthesis were differentially decreased in transgenic mice compared with wild-type mice with fold change from -1.0 to -1.7 at P < 0.005. To reduce potential technical variability of the microarray analysis, we employed qRT-PCR and validated the expression profiles of 33 genes selected from different functional categories with the RNA samples used for microarray analysis, as well as extended muscle tissue samples (Figure 5). The results from all the analyzed genes by qRT-PCR support the significant differences of mRNA expressions detected by microarray analysis although the relative fold change of transgenic mice to wild-type mice did not match exactly. The results from microarray analysis and qRT-PCR showed the same patterns for either differentially increased or decreased expression of the genes, suggesting the reliability of the microarray analysis.

**Table 3:** Differentially expressed genes in myostatin propeptide transgenic mice compared with wild-type littermates

**Accession No. (UniGene)**	**Gene symbol**	**Gene name**	**Transgenic verse wild-type littermates**	
			**-Fold change**	***P*-value**
**Genes related to myogeneis**				

Mm.16528	*Myog*	Myogenin	2.2	0.000244
Mm.22763	*Fstl1*	Follistatin-like 1	2.3	0.000244
Mm.34446	*Cdkn1a*	Cyclin-dependent kinase inhibitor 1A	3.4	0.00002
Mm. 6710	*Rock1*	Rho-associated coil-containing protein kinase 1	3.5	0.00045

**Genes of extracellular matrix components**				

Mm.22621	*Col1a1*	Procollagen, type I, alpha 1	3.7	0.000244
Mm.4482	*Col1a2*	Procollagen, type I, alpha 2	3.4	0.000244
Mm.147387	*Col3a1*	Procollagen, type III, alpha 1	3.6	0.000244
Mm.10299	*Col5a2*	Procollagen, type V, alpha 2	1.9	0.000244
Mm.30477	*Col5a3*	Procollagen, type V, alpha 3	2.1	0.001953
Mm.2509	*Col6a1*	Procollagen, type VI, alpha 1	1.6	0.000244
Mm.1949	*Col6a2*	Procollagen, type VI, alpha 2	2.1	0.001221
Mm.7562	*Col6a3*	Procollagen, type VI, alpha 3	1.9	0.000244
Mm.193099	*Fn1*	Fibronectin 1	2.1	0.001221
Mm.2608	*Bgn*	Biglycan	2.2	0.000244

**Genes related to protein degradation**				

Mm.20863	*Capn3*	Calpain 3	-1.5	0.000732
Mm.24778	*Capn7*	Calpain 7	-1.5	0.004443
Mm.201535	*Casp7*	Caspase 7	-1.7	0.003759
Mm.40986	*Usp25*	Ubiquitin specific protease 25	-1.5	0.002392
Mm.40466	*Fbox32*	Atrogin 1/F-box protein 32	-1.6	0.000244
Mm.24529	*Ube2d3*	Ubiquitin-conjugating enzyme E2D 3	-1.5	0.000732
Mm.78312	*Wwp1*	WW domain containing E3 ubiquitin protein ligase	-1.3	0.000732
Mm.18472	*Psmc6*	Proteasome 26S subunit, ATPase, 6	-1.0	0.001709
Mm.21963	*Psme4*	Proteasome activator subunit 4	-1.2	0.000244

**Genes for mitochondrial ATP synthesis**				

Mm.5545	*Ndufaf1*	NADH dehydrogenase 1 alpha subcomplex, 1	-1.2	0.001953
Mm.41926	*Ndufa4*	NADH dehydrogenase 1 alpha subcomplex, 4	-1.4	0.000244
Mm.27677	*Ndufa5*	NADH dehydrogenase 1 alpha subcomplex, 5	-1.3	0.000244
Mm.29513	*Ndufa7*	NADH dehydrogenase 1 alpha subcomplex, 7,	-1.5	0.000244
Mm.218595	*Ndufas1*	NADH dehydrogenase Fe-S protein 1	-1.2	0.000732
Mm.43162	*Uqcr*	Ubiquinol-cytochrome c reductase (6.4 kD) subunit	-1.1	0.000244
Mm.548	*Cox6c*	Cytochrome c oxidase, subunit VIc	-1.0	0.000244
Mm.29512	*Atpaf1*	ATP synthase mitochondrial F1 complex assembly factor 1	-1.1	0.000585
Mm.41	*Atp5o*	ATP synthase, H+ transporting, mitochondrial F1 complex, O subunit	-1.4	0.000244
Mm.1061	*Fdx1*	adrenodoxin	-1.1	0.000732
Mm.26949	*Etfa*	Electron transferring flavoprotein, alpha polypeptide	-1.1	0.000244

**Carrier proteins**				

Mm.10661	*Slc2a4*	Facilitated glucose transporter 4/solute carrier 2	1.3	0.000244
Mm. 4312	*Slc9a1*	Solute carrier family 9, member 1	3.5	0.000889
Mm. 129110	*Slc7a2*	Solute carrier family 7, member 2	2.1	0.00009
Mm. 28937	*Slc39a4*	Solute carrier family 39, member 4	3.8	0.00002
Mm. 22260	*Slc38a4*	Solute carrier family 38, member 4	1.6	0.00003
Mm. 46067	*Slc25a30*	Solute carrier family 25, member 30	3.1	0.00002
Mm. 19325	*Slc162a9*	Solute carrier family 16, member 9	2.4	0.00448
Mm. 28506	*Slc15a4*	Solute carrier family 15, member 4	1.5	0.00002
Mm. 63479	*Slc15a4*	Solute carrier family 15, member 2	2.3	0.00017

**Others**				

Mm.221164	*Myh6*	Myosin heavy polypeptide 6	1.7	0.00002
Mm157026	*Rsn*	Restin	2.3	0.00002
Mm.17306	*Tmp3*	Tropomyosin 3, gamma	1.4	0.00077
Mm.203875	*Ablim1*	Actin-binding LIM protein	3.3	0.00002
Mm.104975	*M6prbp1*	Mannose-6-phosphate receptor binding protein 1	1.5	0.00003
Mm.26053	*Pfkfb3*	6-phosphfructo-2-kinase/fructose-2,6-biphosphastease 3	2.4	0.00002
Mm.200770	*Gsk3b*	Glycogen synthase kinase 3 beta	1.4	0.00002
Mm. 36640	*Map3k6*	Mitogen activated protein kinase kinase kinase 6	2.2	0.00002

### Enhanced Expressions of Myogenic Regulatory Factors and Extracellular Matrix Components

Myogenesis during embryo development is regulated by myogenic regulatory factors, including MyoD, Myf5, myogenin, and Mrf4. Adult muscle regeneration also requires coordinated responses involving satellite cells and interactions between inductive signals from myogenic factors and the extracellular matrix (ECM). Notably, the expression of myogenin was significantly up-regulated in transgenic mice as determined by microarray analysis (Table 3), which was further confirmed by the qRT-PCR validation. Additionally, cyclin-dependent kinase (Cdk) inhibitor 1A or p21 was also up-regulated by 3.4 fold change by the transgene. Follistatin-like 1 (Fstl1) and Rho-associated kinase (Rock1) showed a fold change of 2.3 and 3.5 by the transgene propeptide, respectively. Interestingly, the results from microarray analysis revealed that several ECM components were up-regulated in transgenic mice, including procollagen type I (α1, α2), III (α1), V (α2, α3), VI (α1, α2, α3), matrix metalloproteinase 2 (MMP2), fibronectin 1 and biglycan (Table 3). The validation analysis by qRT-PCR supports the consistent pattern of the up-regulation of ECM components (Figure 5). The combination of up-regulated myogenic regulatory factors and ECM components supports active muscle fiber fusions still occurring in the one-year-old skeletal muscle of the transgenic mice.

### Down-regulated Expression of Protein Degradation and ATP synthesis

The results from microarray analysis disclosed a large pool of genes that regulate muscle protein deposition. In particular, a group of genes responsible for myofibril protein degradation were significantly down-regulated in transgenic mice, including calpain 3 and 7, caspase 7, ubiquitin (Ub) specific protease 25, Ub-conjugating enzyme E2D3, WW domain containing E Ub protein ligase, proteasome 26S subunit 6, proteasome activator subunit 4 (Table 3). The fold change ranged from -1.0 to -1.7. The expression levels of these genes between transgenic and wild-type mice were further verified by qRT-PCR. The addition of ubiquitin to protein during post-translational modification is a key step in protein degradation, which is dynamic and reversible process controlled by Ub-conjugating and deubiquitylating enzymes. The conjugation of Ub to protein is catalyzed by the successive actions of three types of enzymes: Ub-activating (E1), Ub-conjugating (E2) and Ub-protein ligase (E3) enzymes. Several protease families participate in the deconjugation of ubiquitin. Ubiquitin-specific protease (USP) is one type of deubiqutylating enzymes, which are cysteine proteases, removing ubiqutin from ubiquitylated substrates to rescue them from degradation by the proteasome. Consistent with the biological functions of Ub activating (E1), Ub-conjugating (E2) and Ub-protein ligase (E3), muscle tissue from the transgenic mice showed decreased expression levels of these genes, along with Ub-specific protease 25 (Usp25), which may suggest that the regulation of Ub-dependent protein degradation were down-regulated in the transgenic mice. Additionally, proteins from proteasome pathway such as 26 subunit and activator subunit 4 were also down-regulated in the transgenic mice. Taken together, these data suggest a distinct pattern of reduced protein degradation of adult muscle build-up by the myostatin propeptide.

**Figure 6:**
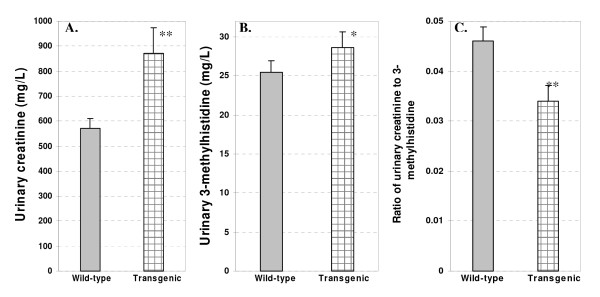
**Urinary creatinine and 3-methylhistidine content**. The mean and SEM was based on 8 mice per group. Urine samples were taken from 12-months-old mice. Statistical differences between transgenic and wild-type mice were determined by two-sided Student's t test (*: P < 0.05; **: P < 0.01).

To confirm the data of muscle protein degradation, we further analyzed urinary creatinine and 3-methylhistidine in the transgenic and wild-type mice at 12 month of age. 3-methylhistidine is formed by post-translational methylation of histidine in muscle actin and myosin. Skeletal muscle mass is the main source of urinary creatinine, which is highly correlated to the total muscle mass of animals fed a meat-free diet [21]. The results showed a significantly increased level of urinary creatinine and 3-methylhistidine in the transgenic mice compared with the wild-type littermates (Figure 6). The ratio of urinary 3-methylhistidine to creatinine, an index of muscle protein catabolism [17], was significantly lower in transgenic mice than that in wild-type mice (P < 0.01, Figure 6C). These results provide further evidence that skeletal muscles in the transgenic mice had a decreased level of protein degradation, consistent with the data extracted from global gene expression analysis by microarray analysis.

Interestingly, the genes related to mitochondrial respiratory chains for ATP synthesis in the transgenic mice were also significantly down-regulated, as represented by consistent lower expressions of NADH dehydrogenase 1α, subcomplex 1, 4, 5, 7 and Fe-S protein1, ubiquinol-cytochorome C reductase subunit, cytochrome c oxidase, ATP synthase, mitochondrial F1 complex factor 1 and O subunit. The fold changes of these genes in transgenic mice compared with wild-type littermate ranged from 1.0 to 1.2. The data suggest that the biochemical activities of oxidative phosphorylation of mitochondrion were down-regulated in the transgenic mice. In myosatin-deficient mice, the ratio of mitochondrial DNA to nuclear DNA and mitochondria number are decreased [22]. We analyzed the enzyme activity of citrate synthase, an exclusive marker of mitochondrial matrix. The muscle tissue had lower citrate synthase activities in transgenic mice than that in wild-type mice (data not shown). As different fiber types contain about same number of mitochondria per myonucleus [22], our data may suggest a decreased number of mitochondria per unit of muscle tissue as a result of enlarged myofiber in the myostatin propeptide transgenic mice. We also noted that increased expression levels of solute carrier proteins such as glucose transporter 4 (Slc2a4) and zinc transporter (Slc39a4) and monocarboxylic acid transporter (Slc16a9, Table 3). Together with decreased mitochondrial ATP synthesis, the data suggest that glycolytic pathway is dominant for glucose metabolism in gastroncmius. Additionally, increased glucose to the muscle may be a significant regulator for muscle protein synthesis as recent studies indicates that glucose alone can also increase protein synthesis in fast-twitch glycolytic muscle [23]. Based on these observations, we believe that enhanced adult muscle build-up by myostatin propeptide create a distinct mechanism that favors efficient ATP synthesis through glycolytic pathway and reduced protein degradation activities to support increase muscle protein accumulation.

## Discussion

In adults, skeletal muscles make up approximately 30 to 40% of body mass. Along with adipose tissue, skeletal muscles growth and maintenance are the main sites of energy utilization. To gain a better understanding adult muscle build-up and its associated energy metabolism, we employed a mouse model with transgenic expression of myostatin propeptide and dramatic muscle growth. The results demonstrated continuous and significant muscle buildup at 12 months of age, with 76% to 153% increase in individual muscles of transgenic mice over the wild-type mice. In muscle tissue, the basal lamina is extremely thin, it is relatively difficult to determine if the nuclei are outside the basal lamina, which can be a good indicator of active satellite cells. In principle, muscle fiber nuclei or "centralized" nuclei can be a good indicator of muscle tissue in active developing stage. From the muscle fiber histology, the transgenic mice also showed the increased nuclei in the central region of muscle fiber, indicating active myofiber fusions in adult stages of the transgenic mice. Previous studies showed that a lack of myostatin increased muscle regeneration through enhanced satellite cell activation and self-renewal, leading to better muscle healing and reduced fibrosis after injury [14,15]. We believe that the enhanced muscle mass of the transgenic mice is initiated by muscle-specific expression of myostatin propeptide at early stage of muscle development. It is continuously maintained until adult stage.

In relation to myogenic regulatory factors, the results from microarray and qRT-PCR analysis showed increased expression of myogenin, Cdk inhibitor p21, Fstl1 and rock1. During myogenesis, MyoD and Myf5 are redundant in myoblast specification whereas myogenin with either MyoD or Mrf4 are required for differentiation [24]. Myogenin is associated with terminal differentiation and fusion of myogenic precursor cells to new or existing fibers. When satellite cells are activated, cell-cycle markers, MyoD and Myf5 transcripts are detectable. Subsequent satellite cell differentiation is marked by the appearance of myogenin [24]. In this study, we did not observe significant changes of MyoD, Myf5 and Mrf4 gene expressions. Cdk inhibitor p21 is induced during early stage of skeletal muscle differentiation, and a high level of p21 expression is sustained when myotubes are re-exposed to high mitogen media, its expression is critical for myocyte viability [25]. Cdk inhibitor p21 was significantly up-regulated in the transgenic mice. Regarding to Fstl1, it is not known about its definite biological role in muscle tissue. Although Fstl1 is classified as a protein similar to follistatin, it only share 7% sequence homology with follisatin [26]. Follistatin binds to activin to neutralize its activity by prevent its binding to type II receptor, therefore blocking myostatin activity when transgenic expression of follistatin specifically to skeletal muscle [5]. Follistatin also directly antagonize myostatin during myogenesis [27]. During the embryonic stage, Fstl1 mRNA is strongly depressed by MyoD induction [28]. In adult muscle, Fstl1 appears to behave like a myokine that acts on vascular endothelial cells as it is secreted into the media by cultured skeletal muscle cells [26]. Fstl1 has direct action on endothelial cell signaling pathways as it was upregulated by muscle ischemia, and its over-expression enhance endothelial cell differentiation and migration. Fstl1 can stimulate revascularization in response to ischemic insult through its ability to activate Akt-eNOS signaling [29]. The increased expression of Fstl1in the adult muscle in the transgenic mouse model may simply result from muscle hypertrophic status. It is certainly worth further investigation. In regard to Rock1, it is a down-stream effector of Rho GTPase or RhoA, playing a critical role in myoblast fusion [30]. RhoA is progressively and specifically down-regulated for execution of tissue-specific morphogenetic events such as fusion into multinucleated syncitia, and maintenance of the terminally differentiated phenotype. Rock appears to concur in keeping myoblasts cycling and in preventing commitment to terminal differentiation [31]. A further study of Fstl1 and Rock1 in this model is likely to yield new information regarding their importance and specific roles in muscle myogenesis in adult stages.

In adults, muscle regeneration requires coordinated actions of capillary morphogenesis, satellite cells, and interactions between inductive signals from myogenic factors and ECM [32,33]. The identified ECM genes such as procollagens had been reported to be up-regulated during adult skeletal muscle regeneration [33]. The increased expression of MMP2 is consistent with a report that demonstrated that increased MMP2 expression and activation is concomitant with regeneration of new myofibers [34]. Biglycan is a leucine-rich proteoglycan involving matrix organization, as well as modulation of growth factors [35]. Biglycan, along with periostin, was highly up-regulated during adult muscle regeneration, and modulated by transformation growth factor β1 [33]. The up-regulation of ECM components further complements the enhanced expression of myogenic regulatory factors of the skeletal muscle. Taken together, these results support a distinct regulatory mechanism of myofiber formation in adult skeletal muscle, resulting from the over-expression of myostatin propeptide.

The balance of protein synthesis and degradation determines net protein deposition of skeletal muscle. In skeletal muscle tissue, four proteolytic systems are possibly involved in protein degradation, including the calpain system, the caspase system, the lysosomal system, and the proteasome. The expression levels of calpain 3, 7 and caspase 7 was down-regulated in the transgenic mice. Caspase 7 is a member of the cysteine-aspartic acid protease family, which plays a central role in the execution-phase of cell apoptosis. Caspases cleave and activate other caspases that subsequently degrade cellular targets, leading to cell death. Caspase 7 is one of the effector caspases, or downstream activator caspases. Skeletal muscles with neurogenic atrophy showed distinct up-regulation of caspase 7 and 9. Expression of caspase 7 was restricted to atrophic fibers, and up-regulated by caspase-9 in proteolytic cascade of degradation of denervated muscle fibers [36]. These results may suggest that enhanced muscle build-up of the adult stage in the transgenic mice is also supported by decreased activities of apoptosis.

It has been well established that myostatin is implicated in the induction of muscle cachexia. Increased levels of myostatin have been implicated in AIDS patients, sarcopenia, chronic human muscle disuse atropy, glucocorticoid-induced muscle atrophy [37-42] A systemic administration of myostatin also induced cachexia [43] while functional blockage of myosatin by intraperitoneal injection of its antibody increases muscle mass and strength in dystrophic mouse model [44]. The mechanism of myostatin action on muscle cachexia appears to activate the ubiquitin proteolytic system independently of cytokine-tumor necrosis factor-α (TNF-α) and transcription factor-κB (NF-κB) pathway [45]. Myostatin induced muscle atrophy through up-regulation of Forkhead box O (FoxO1) transcription factor, and atrophy-related genes such as atrogin-1. FoxO1 have recently beenidentified as a key activator of the atrophy process downstreamof AKT in the IGF-1 signaling pathway [46,47]. Transgenic over-expression of active FoxO1 in skeletal muscle was also shown to inhibit protein synthesis, causing severe skeletal muscle atrophy [48]. In the transgenic mice with possibly depressed myostatin function by its propeptide, we detected a decreased level of atrogin-1 expression. No significant changes in TNF-α and NF-κB expressions were detected in the muscle tissues from the transgenic mice, which is consistent with the observations of myostatin-knockout mice [45]. The role of Fox O1 in the current myostatin propeptide transgenic model is under investigation.

## Conclusion

The results from microarray analysis of global gene expression profile, supported by qRT-PCR assays and biochemical analysis, provide distinct gene expression patterns that integrate low protein degradation and ATP synthesis for efficient muscle build-up and energy utilization. Adult muscle build-up is sustained by high-level expressions of myogenin, Cdk inhibitor P21, follistatin-like factor (Fstl), and Rho-associated kinase (Rock1), and ECM components such as procollagen, fibronectin and biglycan. Decreased levels of protein degradation and mitochondrial ATP synthesis were coordinately observed in the transgenic mice, suggesting efficient energy utilization for adult muscle build-up. Although the profile change and patterns of muscle gene expressions were caused and maintained by the manipulation of a single gene namely myostatin, many genes associated with myofiber fusions and energy utilizations were dynamically changed accordingly. Therefore, we conclude that adult muscle build-up through decreased protein degradation and mitochondrial ATP synthesis may represent an important mechanism or metabolic type of healthy muscle in adult stages. Given the fact that adult muscle build-up is complicated by age-induced muscle atrophy, we have begun to define more specific mechanisms of myogenic initiation, maintenance of myogenic states and mitochondrial energy production in adult stages. Further studies with this model may shed light on its potential application to the treatment of muscle dystrophy and cachexia by depressing myostatin activity.

## Authors' contributions

BZ and JY conceived and implemented the experimental design. BZ conducted RNA preparations, labeling and microarray data analysis, qRT-PCR analysis and interpreted the results. JY drafted the manuscript. EJL conducted the immunohistochemistry and muscle fiber histology studies. RJW was responsible for generation of the transgenic mice and critically read the manuscript. All authors read and approved the final manuscript.
